# Therapeutical and Pharmaceutical Notes

**Published:** 1899-09

**Authors:** W. J. Martin

**Affiliations:** Kankakee, Ill.


### THERAPEUTICAL AND PHARMACEUTICAL NOTES.

UNDER THE DIRECTION OF W. J. MARTIN, M.D.C.,

KANKAKEE, ILL.

CONSTITUENTS OF DIGITALIS LEAVES. The *Journal of Pharmacology* publishes an article by Cloetta on "The Constituents of Digitalis Leaves," translated by O. Hensel, which gives some interesting details of the amount of glucosides found in this part of the plant. Heretofore most of the exact work on digitalis has been done on the seed, but Cloetta studied the leaves, since he believed that the full therapeutic effects were derived from an infusion of the leaves. After a complete chemical study he concludes that the constituents of the leaves are about the same as those of the seeds; they contain digitonin, digitoxin, and digitalin and the peculiar coloring-matter found in the seed. The digitalin of the seed was not found in the leaves. There is a difference, however, in the quantities of these glucosides in the leaf, even if there is no marked difference in the constituents themselves. In the seeds digitalin represents the active ingredient, while digitoxin is found in small amounts only, whereas in the leaf the reverse occurs. Therapeutically this fact is of moment, since digitoxin is five times as strong as digitalin. The author further brings out the fact that it is almost impossible to separate digitonin from digitoxin, and he has prepared a soluble preparation of digitoxin in digitonin, which can be used hypodermatically and can probably clear up some of the difficulties connected with the pharmacological study of this drug.—*Medical Standard.*

MEDICAMENTS WHICH AGE AFFECTS. Most of the official *Pharmacopœia* preparations are so compounded as to prevent any marked deterioration (*Pacific Medical Journal*), but there are a few which age renders inert or markedly diminished in quality. Tincture of iodine (*U. S. Pharmacopœia*), under careful handling, deteriorates after two months, and should, in consequence, never be prepared in large quantities. Spirits of nitrous ether remain at a fair strength for three months only. Dilute hydrocyanic acid loses half its strength in six months. Among others which deteriorate might be mentioned syrup of wild cherry, syrup of althea, camphor-water, fennel-water, anis-water, dilute nitrohydrochloric acid, etc.—*Medical Standard.*

Drugs which Should Not be Dispensed in Capsule Form. The following drugs are hygroscopic, and, therefore, cause the capsule to become softened: Acid phosphate and glycophosphate, sodium bromide, crystallized calcium chlorate, strontium chloride, piperazin, lysidin, chloral, the dry extracts of plants, and in general those preparations produced by evaporation in vacuum. Some solid drugs, when mixed, tend to deliquesce—mixtures of antipyrin and sodium salicylate, for example, when exposed to the air, become altered, or at least discolored, by the contained oxygen. Of this group are the salts of iodine, the alkalies, and the alkaline earths; also aristol. Free iodine combines with the starch of the capsule, producing a bluish-black discoloration.—*Ibid.*

Decomposition of Chloroform. Chloroform is rapidly decomposed when its vapor comes in contact with an exposed gaslight, evolving peculiar chlorine vapors, which are exceedingly irritating to all present, and may be even dangerous to the patient. Chloroform, therefore, should never be administered by gaslight unless the latter be well protected by a closed glass case furnished with abundant provision for the rapid escape therefrom of all decomposed gases.

Fluid Extract of Hyoscyamus. Fluid extract of hyoscyamus frequently deposits crystals of potassium nitrite—a salt always found in good hyoscyamus leaves. It is not a necessary element in the therapeutic efficiency of the preparation, and when found can be removed either by decantation or by filtration with proper precaution.—*Squibbs.*

Tenaline. This is a mixture of the alkaloids of areca nut, which has been successfully experimented with by Dr. Hobday in veterinary practice as a tænifuge. The dose is 6 centigrammes per 500 grammes weight of the animal to which it is administered.

Law's Sheep Dip. ℞.—Tobacco, 16 pounds; oil of tar, 3 pints; soda-ash, 20 pounds; soft-soap, 4 pounds; water, 50 gallons. Steep the tobacco in three successive portions of water and add the other ingredients to the liquor.

Pancreatic Secretion. The introduction of mustard or pepper into the stomach of a rabbit caused the secretion of pancreatic juice to be trebled and even quadrupled. This accounts for the stimulating effects of these condiments upon digestion.

KAISER'S SHEEP DIP. ℞.—Tobacco, 13½ pounds; soda, 3 pints; freshly slaked lime, 4 pounds; soft-soap, 8 pounds; crude carbolic acid, 4 pounds; water, 60 gallons. Infuse the tobacco in the water, strain, and to the infusion add the remaining ingredients.—*Canada Pharmaceutical Journal.*

GRAFTS OF LIVING BONE. Ricard reports two successful attempts to graft living bone. In *Modern Medicine*, February 2, 1898, he reports a case in which the ilium of a dog was engrafted in the place of a portion of the frontal bone (of a man) which had been removed for sarcoma. The second case was that of transplanting an osteoplastic flap from the frontal bone to the nose to restore a syphilitic saddle-nose.

DOG SOAP. Heat together in a water-bath 5 parts of petroleum (crude oil), 4 parts of wax, and 5 parts of alcohol. When a homogenous mixture is obtained add 12 to 15 parts of ivory or other good soap, cut into fine shavings, and stir vigorously until dissolved. When the solution is complete pour into moulds. The resultant soap has a strong odor of petroleum, and is said to be very efficacious, if not in killing, at least in driving away, fleas and other vermin. The soap can be used on the human skin as well.

PREPARATION OF TINCTURE OF IODINE. In the preparation of tincture of iodine designed for veterinary use we have found that wood spirit (methylic alcohol), as a substitute for ethylic or grain alcohol, to be of decided advantage. Not only is the wood spirit much cheaper than the grain alcohol, but the solvent properties of the wood spirit upon the iodine crystals is much greater than the grain alcohol. By the formation of methyl-iodide in the tincture of iodine prepared with wood spirit the vesicatory and absorbent properties of the tincture are much increased. As a basis in the preparation of the various liniments used in veterinary practice, and also of extemporaneous prescription for external use, wood spirit answers admirably in place of the much more expensive grain alcohol. Wood spirit is also a very quick solvent for bichloride of mercury, and hence will be found of decided advantage in preparing the many different forms of solutions in which the drug is used in veterinary practice. On account of its toxic properties wood spirit should never be used internally.

CORK-STOPPERED bottles are superior to glass-stoppered ones in retaining chloroform in stock-bottles.

Cerate of Cantharides. The active principle of cantharides is not soluble in water, and, therefore, a thin film of water will prevent the blistering preparation coming in contact with the cuticle. Both the healthy and unhealthy secretions of the skin are liable to so cover it as to prevent the necessary close contact, and even soap and water, unless very thoroughly applied, do not always remove these excretions; but the excretions which are not removed by soap and water are easily soluble in dilute acetic acid or vinegar, while the active principle of cantharides is also freely soluble in acetic acid, and hence the utility of the old-fashioned mode of cleaning the surface before blistering.—*Squibb's Price-list.*

Compound Tincture of Opium. ℞.—Tr. opii, ℥ij; tr. capsici, ℥ij; sp. camphoræ, ℥ij; chloroform, ʒvj; sp. vini rect., ℥ij.—M. Sig.—Dose for an adult horse, from one and a half to two ounces in a half-pint of warm water. It is an excellent anodyne and carminative in flatulent or spasmodic colic in the early stages and also in diarrhœa, etc. The additions of small doses of the fluid extract of nux vomica to the above prescription will be of decided value in cases of acute indigestion.

Tincture of Chloride of Iron. Tincture of chloride of iron is not fit to use until at least six months old, and much better still if one year old. An important part of the therapeutic value of this tincture depends upon ethers that are slowly generated, and the essential properties of an old as compared with a recently made tincture are very markedly evident upon examination.

Scab in Sheep. In an English agricultural journal Prof. Axe deals with sheep scab. He says that the scab acarus is an egg-producing creature, which deposits its eggs upon the skin of sheep. The effectual remedy for killing these parasites is the ordinary process of dipping in a poisonous solution. This, however, does not effect a complete cure, as the eggs, with their impermeable shell, are in nowise injured, and they in due course of time hatch in the ordinary way, and the skin will again swarm with parasitic life. If, therefore, the disease is to be thoroughly eradicated the sheep must again be dipped before the newly born acari have acquired the power of reproduction, or they, as their parents did before them, will deposit eggs which will be hatched after the second application. Not more than fourteen days should be allowed to elapse between the first and second dippings.

TO PROTECT CATTLE FROM FLIES. The *Suddentsche Apstheker Zeitung* gives the following formula for a preparation for keeping flies off of cattle: ℞.—Oil of cloves, 3 parts; oil of bay, 5 parts; tincture of eucalyptus, 5 parts; alcohol, 150 parts; water, 200 parts. M.—*Druggists' Circular.*

CAMPHOR AS AN ANTIDOTE TO CARBOLIC ACID. Dr. Alvarez (*Zeitschr. f. Pharm.*) has successfully used camphor in a serious case of poisoning with carbolic acid, after having accidentally noted the good effects of camphorated oil on burns caused by carbolic acid. In the above-mentioned case (an attempt at suicide) 100 grammes of camphorated oil was administered. Within an hour the patient was much improved, and in a short time recovered completely.

DEADLY "HEADACHE POWDERS." Judging from the mortuary lists published in the medical journals, the deadly "headache powder" is reaping a rich harvest of victims. These powders usually contain acetanilid or other active coal-tar products, and are prepared in a ready and convenient form for self-administration. The indiscriminate use of such active drugs without proper medical supervision cannot fail to have a most deleterious effect upon the general health of the individual partaking of them.

PYOKTANIN: METHYL-VIOLET. Originally designed for dyeing purposes, this chemical is now largely used as a therapeutic agent. It is a powerful antiseptic, and the claim is made for it that it can be applied to the most delicate mucous membrane without producing irritation. As a germicide and antiseptic, pyoktanin stands high in the list. It destroys the vitality of anthrax bacilli in solution of 1 : 1000, and retards the development of pus cocci in solution of 1 : 2000. Pyoktanin, when applied to an inflamed mucous membrane, stains the same an intense blue; this color remains for a number of days, and, of course, the pyoktanin is active as an antiseptic as long as any color remains.

SOLUTION FOR ECZEMA. Picric acid, 3 parts; tepid boiled water, 250 parts. Mix and apply over diseased surface.

The following solution of picric acid has been used in France in human practice in suppuration of the internal ear, and might be used with equally good results in that obstinate affection, canker of the dog's ear: Picric acid, 3 grains; alcohol, 45 minims; distilled water, 300 minims.

